# Health-related quality of life of older adults residing in social housing: a cross-sectional descriptive study using the EQ-5D-5L

**DOI:** 10.1186/s41687-026-01041-0

**Published:** 2026-04-08

**Authors:** Gina Agarwal, Sahar Popal, Francine Marzanek, Ricardo Angeles, Melissa Pirrie, Christie Koester, Jasdeep Brar, Mikayla Plishka, Guneet Mahal, Manasvi Vanama

**Affiliations:** 1 Department of Family Medicine, McMaster University David Braley Health Sciences Centre, 5th Floor 100 Main Street West, L8P 1H6 Hamilton, ON Canada; 2 Department of Health Research Methods, Evidence, and Impact, McMaster University, 1280 Main Street West, Hamilton, ON L8S 4L8 Canada

**Keywords:** Health-related quality of life, Older adults, Social housing, Low-income

## Abstract

**Background:**

This paper addresses the critical knowledge gap of describing and examining the Health-Related Quality of Life (HRQoL) of older adults living in social housing, highlighting their unique health challenges often overlooked in broader Canadian norms.

**Methods:**

This study conducted a cross-sectional analysis of data from participants in the Community Paramedicine at Clinic (CP@clinic) Program from 2019 to 2024. HRQoL was assessed using the EuroQol five-dimension five-level (EQ-5D-5L) instrument. Descriptive statistics summarized EQ-5D-5L measures (dimensions, utility scores, EQ-VAS) across age groups and sex. Bivariate tests were conducted to examine the association between demographic factors and HRQoL outcomes.

**Results:**

A total of 2286 participants were included, with 86.6% reporting problems in at least one EQ-5D-5L dimension. The mean EQ-5D-5L utility score was 0.771 (SD = 0.176), while the mean EQ-VAS score was 69.58 (SD = 20.577). Utility scores were highest among participants aged 75 and older (0.781) and lowest in the 55–64 age group (0.723). Participants with at least a high school diploma, those living with others, and those with a partner had significantly higher utility scores.

**Conclusions:**

This study provides important insights into the HRQoL of low-income older adults in social housing, a group that remains underrepresented in existing research. The findings can help inform policies and interventions aimed at improving overall well-being and quality of life in this setting.

## Abstract (plain language summary)

Older adults living in social housing often face a combination of issues, including low income, ongoing health problems, and limited access to healthcare. These factors can have a significant impact on both their physical and mental well-being. Despite this, there is little research focused on their quality of life. Our study aimed to better understand the unique health-related challenges faced by older adults in this setting. Using the EQ-5D-5L tool, we assessed the health-related quality of life of older adults in social housing and compared it to Canadian population norms. We found that the health-related quality of life scores for older adults in social housing were notably lower, with the lowest scores seen in those aged 55 to 64. These results highlight a critical need for targeted health services and community supports. Addressing the broader social determinants of health, such as income and housing, is necessary to address the needs of this population.

## Introduction

Quality of life (QoL) encompasses an individual’s emotional, social, and physical well-being, and ability to carry out daily activities [1]. It is assessed using specialized instruments that evaluate both functional abilities and personal perceptions of illness [2, 3]. These tools provide valuable insights into the impact of health, disease, and social environments from the patient’s perspective [4]. They can also help track changes in well-being over time [5, 6]. One widely used measure is the standardized EuroQol five-dimension (EQ-5D) instrument, which measures health-related quality of life (HRQoL) on five dimensions: mobility, self-care, usual activities, pain/discomfort, and anxiety/depression [7]. The EQ-5D-5L version of the instrument provides five levels of severity as the response options for each dimension. Additionally, it features a Visual Analogue Scale (EQ-VAS) that allows respondents to rate their current health on a scale from 0 to 100 [7].

HRQoL analyses are useful for understanding the holistic impact of diseases and treatments on individuals. Research indicates that QoL varies by demographic factors such as age [8, 9]. A study on Canadian population norms for the EQ-5D-5L found that individuals aged 55–64 reported the lowest HRQoL scores [10]. Similarly, research has shown that HRQoL among community-dwelling older adults is strongly influenced by a combination of health, social, and contextual variables [11]. Globally, lower income levels are consistently associated with poorer health outcomes and reduced HRQoL [12]. However, studies on HRQoL in older adults residing in social housing remain limited.

Older adults constitute a significant portion of social housing residents, and over one-third of individuals on the waiting lists are aged 65 or older [13, 14]. Social housing, also referred to as community or public housing, provides government-supported, affordable rental accommodations for households with low-to-moderate incomes [15]. Recognized as a key social determinant of health, social housing is often associated with adverse health outcomes [16, 17]. Older adults in this setting often experience intersecting challenges of advanced age, a high prevalence of chronic illnesses, and limited financial resources [14, 18]. Furthermore, in our sample, most participants were women, many of whom are widowed and living alone, and educational attainment is generally lower in this setting [19]. These demographic and housing characteristics guide our conceptual model for the study: age, sex, education, living arrangement, and the housing environment are expected to relate to HRQoL by shaping social support, access to resources, and exposure to health risks within this population.

Despite the increasing size and the structural challenges many face, research on the HRQoL of low-income older adults living in social housing remains limited, and existing studies differ in scope and focus from the current paper. Addressing this gap is critical to understanding their health needs and informing strategies for healthcare resource allocation and system improvements. Accordingly, the objective of this study was to evaluate the HRQoL of older adults residing in social housing, and to describe the relationship between HRQoL, age, sex, education and living arrangements within this population.

## Methods

### Setting

This cross-sectional study collected data from participants of the Community Paramedicine at Clinic (CP@clinic) program, an established community-based, paramedic-led health and wellness program in social housing in Ontario [20]. CP@clinic is a weekly, drop-in program designed to promote health and prevent chronic disease among community-dwelling, low-income older adults. The program aims to identify modifiable health risk factors, provide education, and connect participants with appropriate community resources. Advertised through posters displayed in residential buildings, the program encourages residents to attend sessions for preventative health assessments. During these sessions, paramedics conduct one-on-one validated health-risk assessments, deliver tailored health education, and facilitate referrals to community resources based on assessment outcomes [21].

### Participants

Data was obtained from the CP@clinic database for program participants who attended the CP@clinic program between 2019 and 2024. The participants were older adults, aged 55 years and over, living in social housing across 24 municipalities in Ontario, Canada. All participants with a complete set of EQ-5D-5L responses were included in this study. A total of 2286 CP@clinic Program attendees were eligible for inclusion in the study analysis out of 2550 attendees (89.6%). All participants provided written informed consent, collected by the paramedics, and the study received ethical approval from the Hamilton Integrated Research Ethics Board (#11078).

### Data collection and measures

As part of the CP@clinic Program, community paramedics collected sociodemographic data (age, sex, education, marital status, and living arrangements), HRQoL, and health-risk assessments using the online CP@clinic database.

The EQ-5D-5L instrument in the CP@clinic database assessed HRQoL dimensions: mobility, self-care, usual activities, pain/discomfort, and anxiety/depression [7]. For each dimension, participants indicated their current health status by selecting one out of five levels: no (1), slight (2), moderate (3), severe (4), extreme (5). For mobility, options ranged from “no problems walking” to “unable to walk.” For self-care, responses ranged from “no problems washing or dressing” to “unable to wash or dress.” For usual activities, options ranged from “no problems” to “unable to perform usual activities.” For pain/ discomfort, options ranged from “no pain/ discomfort” to “extreme pain or discomfort.” For anxiety/ depression, options ranged from “not anxious or depressed” to “extremely anxious or depressed [7].” To clearly distinguish whether or not participants reported experiencing any problems, the five-level response scale was dichotomized into “no problems” (level 1) and “any problems” (levels 2–5).

The EQ-5D-5L responses were summarized using a five-digit code, where “11111” indicated the optimal health state (no issues in any dimension) and “55555” reflected the most severe health state (extreme issues in all dimensions). Health State Utility Values were assigned to these health states, with values ranging from − 0.148 for the worst health state (55555) to 0.949 for the best health state (11111) using the Canadian value set derived from data obtained from 1,073 members of the Canadian general population [22]. The EQ-5D-5L also incorporates the EQ Visual Analogue Scale (EQ-VAS), which captures participants’ self-assessed health status on a scale ranging from 0 (representing the worst imaginable health) to 100 (representing the best imaginable health). This component quantitatively measures health outcomes based on the individual’s personal evaluation [7].

### Data analysis

Descriptive statistics, including proportions, means, and standard deviations, were calculated to characterize the study population. Frequency and proportions were used to summarize responses to each EQ-5D-5L dimension by age category. For the continuous EQ-5D-5L utility score, mean, and standard deviations were analyzed by sex, age category, education status, marital status, living alone (yes/no), and ethnicity. Ethnicity was categorized as White and non‑White; the non‑White group included participants identifying as Aboriginal, Black (Afro‑Caribbean), East Asian (e.g., Chinese, Vietnamese, Filipino, Korean), South Asian (e.g., East Indian, Pakistani, Sri Lankan), and Latin American, Arab, or West Asian.

Exploratory bivariate analyses were conducted to assess the associations between these socio-demographic characteristics and both EQ-5D-5L utility scores and EQ-VAS scores. The Shapiro-Wilk test was applied to evaluate the normality of the utility and EQ-VAS score distribution. If the data followed a normal distribution, parametric tests (t-test and ANOVA) were employed; otherwise, the Wilcoxon-Mann-Whitney test was used. These analyses were descriptive in nature and were not intended to establish causal relationships and subgroup comparisons should be interpreted as exploratory rather than definitive, given the absence of adjustment for potential confounders. Results were considered statistically significant if two-sided p-values were less than 0.05. All analyses were performed using IBM SPSS Statistics version 29.0.

Missing data were assessed for the EQ‑5D‑5L and EQ‑VAS measures, and complete‑case analysis was used. To evaluate potential bias, we compared the sociodemographic characteristics of attendees excluded due to incomplete EQ‑5D‑5 L data with those included in the study. EQ‑VAS analyses were further restricted to the 2,186 participants with complete EQ‑VAS scores.

## Results

This study included a total of 2286 participants, comprising 516 men (24.2%) and 1,614 women (75.8%). The mean age of the participants was 77.0 years (SD = 8.87), with ages ranging from 55 to 99 years. The demographic profile indicated that the majority of participants were white (76.2%), lived alone (74.0%), had a high school education or less (60.3%), and were either widowed, divorced, separated, or single (72.7%). An analysis of the socio-demographic profiles of the 264 individuals excluded due to missing EQ-5D-5L data, revealed no significant differences compared with the 2,286 included in the study. This suggests that the missing data are unlikely to have introduced bias into our findings. Detailed demographic characteristics of the participants are presented in Table 1.

**Table 1:** Demographics and descriptive characteristics of study participants

*N*, %	Men	Women	Total
	516 (24.2)	1614 (75.8)	2130*
Age, mean (SD)	75.9 (8.90)	77.5 (8.75)	77.0 (8.87)
Age group, n (%)			
55–64	59 (11.4)	119 (7.4)	178 (8.4)
65–74	166 (32.2)	483 (29.9)	649 (30.5)
75+	291 (56.3)	1012 (62.7)	1303 (61.2)
Education, n (%)			
Some High School or less	153 (29.7)	630 (39.0)	783 (36.8)
High School Diploma	122 (23.6)	378 (23.4)	500 (23.5)
Some College/ University	241 (46.7)	606 (37.5)	847 (39.8)
Marital Status, n (%)			
Married	193 (37.4)	229 (14.2)	422 (19.8)
Common-law	6 (1.2)	15 (0.9)	21 (1.0)
Widowed	81 (15.7)	696 (43.1)	777 (36.5)
Separated	29 (5.6)	84 (5.2)	113 (5.3)
Divorced	101 (19.6)	337 (20.9)	438 (20.6)
Single, Never Married	75 (14.5)	144 (8.9)	219 (10.3)
Ethnicity, n (%)			
White	357 (69.2)	1266 (78.4)	1623 (76.2)
non-White	144 (27.9)	309 (19.1)	453 (21.3)
Living Alone, n (%)			
Yes	284 (55.0)	1291 (80.0)	1575 (74.0)
No	215 (41.7)	294 (18.2)	509 (41.4)

*156 participants did not report their sex, or it was missed in recording by paramedics at entry

### EQ-5D-5L dimensions

All participants had completed responses for the EQ-5D-5L assessments, detailed in Table 2. Of the total participants, only 307 (13.4%) reported no problems in any of the five dimensions. The proportion of respondents reporting no problems was similar across age groups: 13.9% among those aged 55–64, and 13.4% in both the 65–74 and 75 + categories. Pain/discomfort was the most commonly affected dimension, with 66.6% of participants indicating having any problems. This was followed by mobility (57.9%), anxiety/depression (48.8%), and ability to do usual activities (39.2%). The self-care dimension had the lowest rate of reported problems at 25.5%. Consistent with these findings, the pain/discomfort and mobility dimensions recorded the largest number of severe and extreme responses.

**Table 2:** Distribution of EQ-5D-5L responses by age groups

	Age Group			
	55–64 *n* (%)	65-74 *n* (%)	75 + *n* (%)	Total *n* (%)
No problems in all 5 dimensions	28 (13.9)	94 (13.4)	185 (13.4)	307 (13.4)
Reported problems in any dimension	173 (86.1)	607 (86.6)	1199 (86.6)	1979 (86.6)
Mobility				
No problems	90 (44.8)	311 (44.4)	561 (40.5)	962 (42.1)
Slight	50 (24.9)	198 (28.2)	432 (31.2)	680 (29.7)
Moderate	42 (20.9)	132 (18.8)	288 (20.8)	462 (20.2)
Severe	14 (7.0)	48 (6.8)	88 (6.4)	150 (6.6)
Extreme	5 (2.5)	12 (1.7)	15 (1.1)	32 (1.4)
Self-care				
No problems	148 (73.6)	542 (77.3)	1015 (73.3)	1705 (74.6)
Slight	31 (15.4)	87 (12.4)	192 (13.9)	310 (13.6)
Moderate	14 (7.0)	51 (7.3)	129 (9.3)	194 (8.5)
Severe	4 (2.0)	15 (2.1)	29 (2.1)	48 (2.1)
Extreme	4 (2.0)	6 (0.9)	19 (1.4)	29 (1.3)
Usual Activities				
No problems	114 (56.7)	452 (64.5)	822 (59.4)	1388 (60.7)
Slight	35 (17.4)	132 (18.8)	302 (21.8)	469 (20.5)
Moderate	35 (17.4)	79 (11.3)	188 (13.6)	302 (13.2)
Severe	13 (6.5)	27 (3.9)	47 (3.4)	87 (3.8)
Extreme	4 (2.0)	11 (1.6)	25 (1.8)	40 (1.7)
Pain/discomfort				
No problems	61 (30.3)	215 (30.7)	489 (35.3)	765 (33.5)
Slight	43 (21.4)	219 (31.2)	457 (33.0)	719 (31.5)
Moderate	69 (34.3)	193 (27.5)	326 (23.6)	588 (25.7)
Severe	23 (11.4)	65 (9.3)	97 (7.0)	185 (8.1)
Extreme	5 (2.5)	9 (1.3)	15 (1.1)	29 (1.3)
Anxiety/depression				
No problems	77 (38.3)	309 (44.1)	784 (56.6)	1170 (51.2)
Slight	53 (26.4)	196 (28.0)	345 (24.9)	594 (26.0)
Moderate	42 (20.9)	152 (21.7)	208 (15.0)	402 (17.6)
Severe	24 (11.9)	34 (4.9)	38 (2.7)	96 (4.2)
Extreme	5 (2.5)	10 (1.4)	9 (0.7)	24 (1.0)

### EQ-5D-5L utility scores

**Fig. 1:**
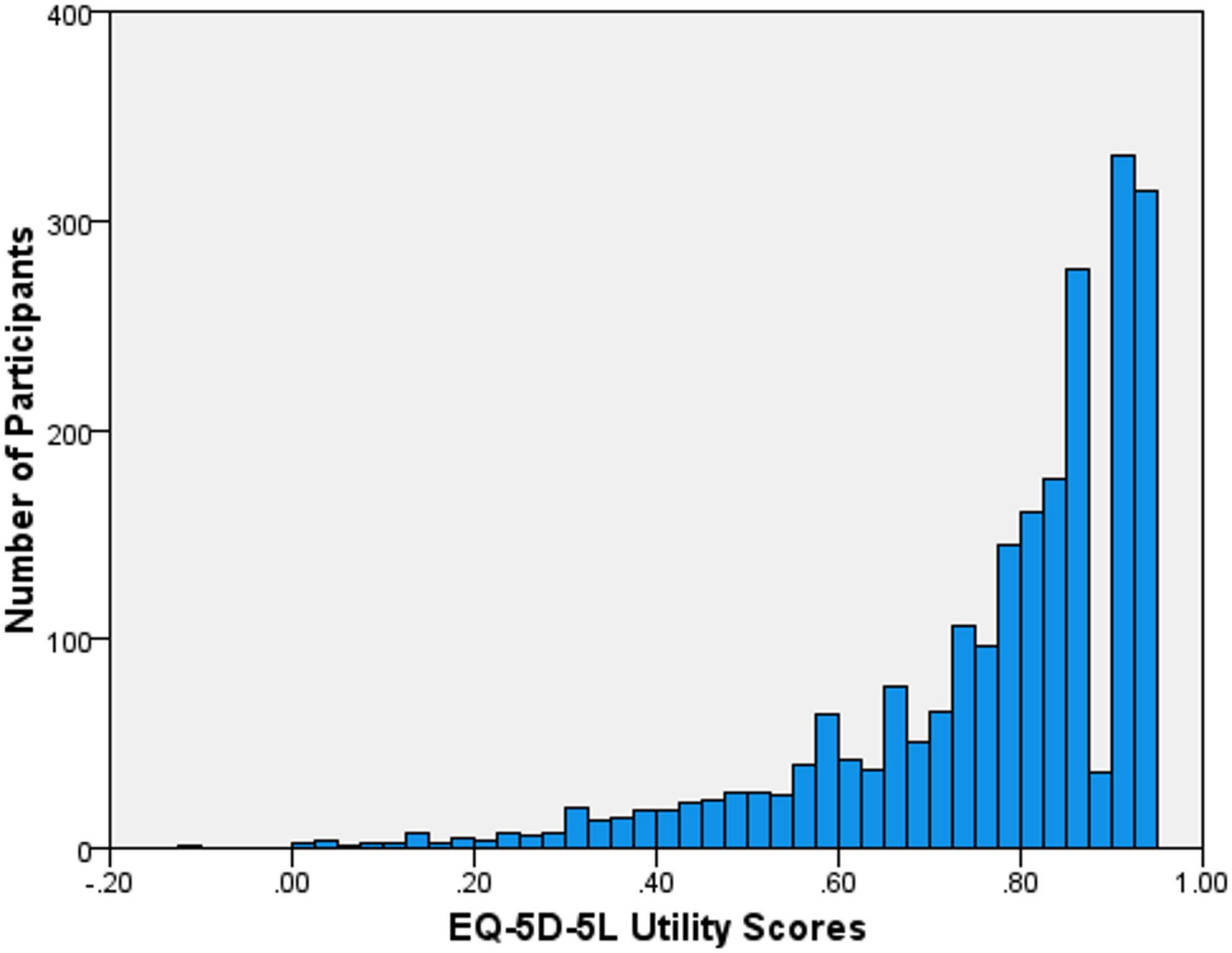
Distribution of EQ-5D-5L utility scores

**Fig. 2:**
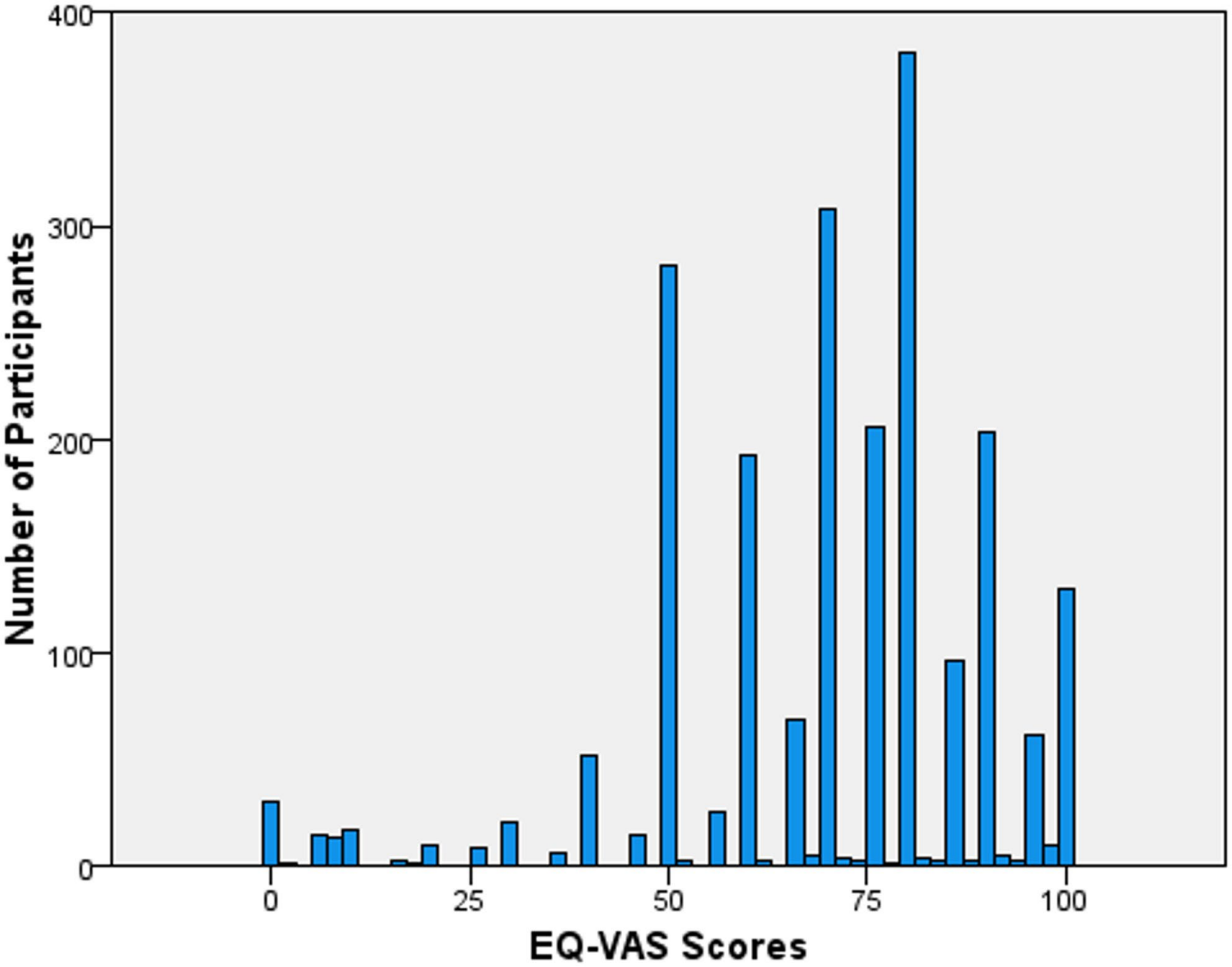
Distribution of EQ-VAS Scores

The mean EQ-5D-5L utility score was 0.771 (SD = 0.176, range − 0.104 to 0.949). Figure 1 displays the distribution of EQ-5D-5L utility scores, and Fig. 2 shows the distribution of EQ-VAS scores. Table 3 presents the mean EQ-5D-5L utility scores by demographic characteristics and sex. The highest overall mean utility score was 0.781 (SD = 0.166) in the 75 + age group, and the lowest was 0.723 (SD = 0.213) in the 55–64 age group.

**Table 3:** Descriptive analysis of EQ-5D-5L utility scores by sociodemographic characteristics

Variable	Total(*N* = 2286) Utility Score Mean (SD)	*p*-value*	Men (*n* = 516/2130) Utility Score Mean (SD)	Women (*n* = 1614/2130) Utility Score Mean (SD)
All	0.771 (0.176)		0.795 (0.166)^a^	0.762 (0.179)^a^
Age group				
55–64	0.723 (0.213)	< 0.001	0.734 (0.207)	0.707 (0.222)
65–74	0.764 (0.179)		0.793 (0.165)	0.756 (0.183)
75+	0.781 (0.166)		0.809 (0.154)	0.772 (0.171)
Education				
Some High School or less	0.763 (0.174)	0.017	0.798 (0.163)	0.754 (0.176)
High School Diploma or Higher Education	0.775 (0.176)		0.794 (0.167)	0.767 (0.181)
Marital Status				
Have a Partner	0.785 (0.183)	< 0.001	0.817 (0.154)	0.758 (0.202)
No Partner	0.766 (0.174)		0.777 (0.175)	0.762 (0.175)
Ethnicity				
White	0.765 (0.176)	< 0.01	0.767 (0.162)	0.780 (0.171)
non-White	0.790 (0.175)		0.833 (0.131)	0.831 (0.150)
Living Alone				
Yes	0.766 (0.172)	< 0.01	0.785 (0.167)	0.760 (0.174)
No	0.788 (0.184)		0.810 (0.161)	0.772 (0.200)

*p values come from the Wilcoxon–Mann–Whitney test for binary variables in the overall sample (i.e., not stratified by sex)

a Sex is statistically significant (*p* < 0.05)

Men had higher mean utility scores across all age groups, with an overall mean of 0.795 (SD = 0.166) compared to 0.762 (SD = 0.179) for women. In both sexes, the highest utility scores were observed in the 75 + age group, with men scoring 0.809 (SD = 0.154) and women 0.772 (SD = 0.171). The lowest scores for both men and women were recorded in the 55–64 age group, with utility scores of 0.734 (SD = 0.207) and 0.707 (SD = 0.222), respectively. The most frequently reported health states were 11111 (13.4%), 11121 (7.3%), 11112 (4.3%), and 11122 (2.5%).

### Associations between sociodemographic characteristics and EQ-5D-5L utility scores

The Shapiro-Wilk test indicated that the EQ-5D-5L utility scores were not normally distributed (*p* < 0.05), so the Wilcoxon-Mann-Whitney test was used for analysis. The results showed that education level, marital status, ethnicity, and living arrangements had a statistically significant association with utility scores. The mean utility scores were lower among individuals with some high school education or less (0.763, SD = 0.174) compared to those with a high school diploma or higher (0.775, SD = 0.176). Similarly, individuals without a partner had lower utility scores than those with a partner (0.766, SD = 0.174 vs. 0.785, SD = 0.183), and those living alone reported lower scores than those living with others (0.766, SD = 0.172 vs. 0.788, SD = 0.184). Additionally, individuals identifying as white (0.765, SD = 0.176) had lower utility scores compared to those from other ethnic backgrounds (0.790, SD = 0.175).

### EQ-VAS scores

The mean EQ-VAS score was 69.58 (SD = 20.577). Table 4 depicts the mean EQ-VAS scores by demographic characteristics and sex. The highest overall mean EQ-VAS score was 70.51 (SD = 20.621) in the 75 + age group, and the lowest was 64.98 (SD = 24.670) in the 55–64 age group, following a similar trend to the mean utility scores.

**Table 4:** Descriptive analysis of EQ-VAS scores by sociodemographic characteristics

Variable	Total(*N* = 2186)^a^ EQ-VAS Score Mean (SD)	*p*-value*	Men (*n* = 492/2037)^b^ EQ-VAS Score Mean (SD)	Women (*n* = 1545/2037)^b^ EQ-VAS Score Mean (SD)
All	69.58 (20.577)		68.09 (21.529)^c^	69.78 (20.488)^c^
Age group				
55–64	64.98 (24.670)	< 0.05	61.42 (26.520)	66.02 (24.255)
65–74	69.07 (18.95)		68.60 (19.187)	68.90 (19.069)
75+	70.51 (20.621)		69.12 (21.559)	70.64 (20.591)
Education				
Some High School or less	69.82 (20.30)	> 0.05	68.46 (23.073)	69.83 (19.791)
High School Diploma or Higher Education	69.44 (20.74)		67.93 (20.870)	69.74 (20.928)
Marital Status				
Have a Partner	67.95 (20.328)	< 0.01	67.21 (21.280)	68.25 (19.835)
No Partner	70.73 (19.689)		69.33 (21.159)	70.78 (19.659)
Ethnicity				
White	70.16 (20.484)	< 0.05	68.35 (22.25)	70.49 (20.14)
non-White	69.19 (17.815)		68.37 (18.464)	69.10 (17.742)
Living Alone				
Yes	70.03 (20.341)	> 0.05	68.22 (21.68)	70.12 (20.34)
No	68.46 (21.254)		67.69 (21.70)	68.6 (21.14)

*p values come from the Wilcoxon–Mann–Whitney test for binary variables in the overall sample (i.e., not stratified by sex)

a 100 participants did not report their EQ-VAS score

b 249 participants did not report sex and/or EQ-VAS scores

c Sex is not statistically significant (*p* > 0.05)

Although women had higher mean EQ-VAS scores across all age groups, with an overall mean of 69.78 (SD = 20.488) compared to 68.09 (SD = 21.529) for men, these results were not statistically significant.

### Associations between sociodemographic characteristics and EQ-VAS scores

The Shapiro-Wilk test indicated that the EQ-VAS scores were not normally distributed (*p* < 0.05), so the Wilcoxon-Mann-Whitney test was used for analysis. The results showed that ethnicity and marital status were significantly associated with EQ‑VAS scores, whereas education and living alone versus with others were not.

The mean EQ-VAS scores were higher among individuals identifying as white (70.16, SD = 20.484) compared to those from other ethnic backgrounds (69.19, SD = 17.815). Similarly, individuals without a partner had higher EQ-VAS scores than those with a partner (70.73, SD = 19.689) vs. (67.95, SD = 20.328).

## Discussion

This study assessed the HRQoL of 2286 older adults residing in social housing who participated in the CP@clinic Program. A substantial majority (86.6%) of participants reported problems in at least one dimension of the EQ-5D-5L. The most frequently reported issues were in the pain/discomfort dimension, followed by mobility, anxiety/depression, usual activities, and self-care. The mean utility score was 0.771 (SD = 0.176), and the subgroup with the highest mean utility score was those aged 75 and older (mean = 0.781, SD = 0.166). The findings of this study provide important insights into an understudied population of low-income older adults living in social housing who often face structural and socioeconomic barriers.

While previous studies have examined HRQoL in broader adult populations, such as those that were used to establish the EQ5D norms in Alberta, Quebec, and Canada, no studies to date have focused on the older adult population residing in social housing [10, 23, 24]. This population faces unique challenges, including the combined effects of aging, chronic illness, and financial hardship, which can significantly impact their well-being [14, 18]. The fact that the current study found that 86.6% of participants reported problems in at least one EQ-5D-5L dimension emphasizes the need for targeted interventions to improve HRQoL in social housing populations, particularly for managing pain and mobility issues, which were the most affected areas.

The mean EQ-5D-5L utility and EQ-VAS score found in the present study (0.771 and 69.6) were lower than those established in the national Canadian norms study (0.864 and 82.3), and the provincial norm studies for Quebec (0.824 and 75.9) and Alberta (0.845 and 77.4) [10, 23, 24]. There were notable additional differences between our findings and the Canadian population norms. Specifically, our study found that far fewer (13.4%) older adults in social housing reported no problems in any dimension, compared to 33.1% in the Canadian norms study. Additionally, participants in the Canadian norms study reported fewer problems across all five dimensions: pain/discomfort (53.1% vs. 66.6% in the current study), anxiety/depression (37.9% vs. 48.8%), usual activities (25.4% vs. 39.2%), mobility (24.1% vs. 57.9%), and self-care (7.6% vs. 25.5%).

However, it is important to note that the Canadian norms study included participants aged 18 and older, whereas our study focused exclusively on individuals aged 55 and above, who may have been expected to have such issues since they were a more aged population. In order to create a more accurate comparison, we have used data from the Canadian norms studies for participants aged 55 and older.^1^ In this comparison, 27.9% of individuals aged 55 years and older reported no problems in any dimension, while the current study in social housing found a rate of 13.4%. This represents a reduction of more than 50% in the proportion reporting full health, highlighting a substantial difference in overall health status. Furthermore, for these older age groups, reported problems in all dimensions were more prevalent than in the full 18 + population, except for the anxiety/depression dimension. Even with this age-matched comparison, individuals in our social housing population reported higher rates of problems across all dimensions: pain/discomfort (58.2% vs. 66.6% in our population), anxiety/depression (31.9% vs. 48.8%), usual activities (31.6% vs. 39.2%), mobility (37% vs. 57.9%), and self-care (9.3% vs. 25.5%). This pattern may reflect underlying socioeconomic and environmental differences that are associated with disparities in HRQoL.

Consistent with findings from the Canadian population norm study, our results showed that the three most commonly reported health states were 11111, 11121, and 11112. The Canadian population norms study also reported that the 55–64 age group had the lowest mean EQ-5D-5L utility score (0.839) [10]. Similarly, our study also identified the 55–64 age group as having the lowest utility score, though it was notably lower at 0.723. Across all age groups, the utility scores in our population were consistently lower than the Canadian norms: 55–64 (0.839 vs. 0.723), 65–74 (0.867 vs. 0.764), and 75+ (0.861 vs. 0.781). These findings suggest that older adults in social housing may face greater health challenges compared to the general population, emphasizing the need for targeted support and interventions. However, it is important to acknowledge that many individuals living in social housing experience low income and chronic health conditions, which act as intersecting factors that may influence overall HRQoL [16]. Given the overlap of these determinants, isolating the specific contribution of each factor is challenging. This observation aligns with prior research indicating that individuals with lower income or chronic conditions consistently report reduced utility scores [23, 24]. Differences in HRQoL among adults aged 55–64 may reflect a range of social and health‑related factors that were not directly measured in this study. This age group often experiences the unique pressures of midlife, where financial and employment demands intersect with caregiving responsibilities and the onset of multiple chronic conditions [25, 26]. These challenges, along with evidence that coping strategies may still be developing in midlife, have been proposed as factors that could influence self‑perceived HRQoL [27, 28]. These interpretations are offered as hypothesis‑generating and warrant further investigation.

Additionally, while the Canadian norms study found that women had lower utility scores than men only in the 65–74 age group among individuals aged 55 and older, our study observed lower utility scores for women across all age groups (55–64, 65–74, and 75+). This pattern suggests that sex-based HRQoL disparities may be more pronounced in populations experiencing socioeconomic and structural barriers, reflecting the broader impact of socioeconomic status on health.

Differences between EQ‑5D‑5 L utility scores and EQ‑VAS ratings are well documented. EQ‑VAS tends to show greater variability because it reflects individuals’ subjective assessments of overall health, whereas utility scores apply societal preference weights that compress values toward the upper end and create skewed distributions [29, 30]. As a result, utility scores often display ceiling effects and negative skew [31, 32]. The greater variability in VAS scores and the negatively skewed utility scores observed in our sample are consistent with these established patterns [33].

The findings of this study should be interpreted in light of its limited generalizability. Participants were older adults living in social housing and enrolled in the CP@clinic Program, a context that differs from the broader older adult population in Canada. Their socioeconomic circumstances, health needs, and access to community‑based supports may not reflect those of older adults in other housing settings or regions. As a result, the HRQoL patterns observed here may not be directly transferable to populations with different demographic or socioeconomic profiles. Nonetheless, the results offer valuable insight into an understudied group and may be informative for similar social housing environments or community‑based health programs.

Based on our findings, pain/discomfort and mobility emerged as the most affected HRQoL domains and should be prioritized in future support efforts. Lower HRQoL was also more evident among adults aged 55–64, those living alone, and individuals with lower educational attainment. These results highlight the need for policies and social services in Ontario that strengthen access to mobility and pain‑management supports and enhance community‑based programming for older adults in social housing who may face social isolation and structural barriers.

Future research should specifically explore how HRQoL is impacted by specific social determinants of health, such as housing conditions and social support. An in-depth investigation of these influences can uncover gaps in support and inform more targeted approaches. Furthermore, insights into strategies that can meaningfully improve quality of life can be provided through evaluations of the effectiveness of existing programs such as community health initiatives, mental health services, and improved access to home modifications or mobility aids. For example, ‘CP@clinic’ is a chronic disease prevention and health promotion program for older adults in social housing that aims to improve their health and quality of life by providing health risk assessments and offering appropriate referrals to community health resources and primary care [20, 21]. Similarly, other Aging at Home strategies have been implemented in various provinces with the aim of supporting older adults to remain independent by offering caregiver support, integrating home care, and conducting home modifications [34]. These programs serve as promising models that might address the complex needs of older adults facing structural barriers and living in social housing.

## Limitations

This study may be subject to self-selection bias, as participation in the CP@clinic Program was available to all building residents, who could decide whether or not to attend, rather than individuals being randomly selected. Individuals with better health and higher quality of life may have been more likely to enroll, potentially limiting the sample’s representativeness and skewing the data to show more positive results. A truly random sample might have reported lower HRQoL. Additionally, reliance on self-reported EQ-5D-5L responses introduces additional potential bias, as the data accuracy is dependent on participants’ comprehension and recall. However, the EQ-5D-5L is a widely used and validated tool. Another limitation relates to the presentation of EQ‑5D‑5L dimension data: responses were dichotomized in the results section solely to report the prevalence of any problems within each domain. This descriptive approach did not affect statistical analyses, as the full five‑level response structure was retained and is presented in Table 2. Lastly, gender data were missing for 156 participants, either due to non-disclosure or recording errors by paramedics during data entry.

## Conclusions

This study offers important insights into the HRQoL of low-income older adults in social housing using the EQ-5D-5L, which shows that their utility scores are lower than those of the age-matched general Canadian population. Future research could examine specifically how social determinants of health, including housing conditions, influence quality of life and evaluate the effect of targeted interventions, such as community health programs, mental health support, and improved access to mobility aids, on health-related quality of life.

## Data Availability

The data supporting this study are not publicly available due to privacy concerns. De-identified data may be provided by the corresponding author upon reasonable request.

## Footnotes

1 This analysis was conducted post hoc and was not presented in the referenced paper. Comparative data were obtained and analyzed from the published Canadian population norms for the EQ-5D-5L.

## Abbreviations

QoL: Quality of Life
HRQoL: Health-Related Quality of Life
CP@clinic: Community Paramedicine at Clinic
EQ-5D-5L: EuroQol 5 Dimensions 5 Levels
EQ-VAS: EuroQol Visual Analogue Scale
